# Public Health Policies on E-Cigarettes

**DOI:** 10.1007/s11886-019-1204-y

**Published:** 2019-08-28

**Authors:** Aditya Bhalerao, Farzane Sivandzade, Sabrina Rahman Archie, Luca Cucullo

**Affiliations:** 1 Department of Pharmaceutical Sciences, Jerry H. Hodge School of Pharmacy, Texas Tech University Health Sciences Center, 1300 S. Coulter Street, Amarillo, TX 79106 USA; 2 Center for Blood Brain Barrier Research, TTUHSC, Amarillo, TX 79106 USA

**Keywords:** E-cigarettes, Electronic cigarettes, Electronic nicotine delivery systems, Policy, Regulation, Public health

## Abstract

**Abstract:**

Tobacco continues to kill about 0.48 million Americans per year and there are currently 34.3 million smokers in the USA. As a consequence of the First Surgeon General’s Report on Tobacco in 1964, tobacco control interventions on part of the government led to a significant decline in conventional tobacco product usage over the last few decades. However, more recently, a new entity in the form of electronic cigarettes has risen rapidly and has exposed a younger population to a plethora of dangerous consequences. Looking at e-cigarettes from the perspective of tobacco control however raises a lot of challenges. There is little doubt that existing smokers of combustible cigarettes who switch to e-cigarettes will be switching to a less harmful product. However, if the younger generation begins using e-cigarettes as a result of targeted marketing, appealing flavors and ‘safer alternative’ perception, decades of progress made in conventional tobacco control will be negated. Governments at the federal, state, and local levels have a mandate to once again implement new public health policies to ensure that non-conventional tobacco products like e-cigarettes are available as smoking cessation tools for existing smokers but at the same time do not play a role in ruining the health of future generations through addiction and disease.

**Purpose of Review:**

To review the present scenario of regulations and policies impacting public health with respect to electronic nicotine delivery systems (ENDS) with the objective of providing a meaningful and balanced view of the challenges at hand with plausible recommendations.

**Recent Findings:**

Nicotine in tobacco is known to cause addiction and dependence. It is particularly potent in children and young adults. E-cigarettes can deliver high concentrations of nicotine, and these concentrations can vary depending on the numerous constituents within the e-cigarette which vary greatly from one another. Use of e-cigarettes is implicated as a risk factor for future cigarette use in young adults. Moreover, e-cigarette usage patterns also depend on several sociodemographic factors. Banning tobacco products has shown to reduce smoking risk in youth and as such, strong e-cigarette regulation measures are needed for prevention.

**Summary:**

Effective regulation of ENDS faces a multitude of challenges. One such challenge is to prevent youth and non-smokers from getting habituated to nicotine through e-cigarettes. The intention of tobacco companies to sustain sales through harmful marketing strategies that tone down the risks and highlight e-cigarettes as a “much safer alternative” while promoting flavors appealing to children should be immediately prohibited. Another hazard is the endorsement of ENDS as devices meant for enhancing social interaction which opens a path for youth to make erroneous choices under peer pressure. On the other hand, several studies have reported that e-cigarettes significantly reduce an existing smoker’s risk of being exposed to toxic tobacco smoke constituents that are normally present in cigarette smoke. This leads to the conclusions that e-cigarettes can be a tool for smoking cessation for current smokers. Public policy must take a multi-dimensional approach to balance these two extremes.

## Introduction

Electronic nicotine delivery systems (ENDS) are commonly known as electronic cigarettes or e-cigarettes and vape pens. These devices are meant to deliver nicotine by heating up a vape liquid into an inhalable aerosol. The vape liquid is a solution containing nicotine, flavoring agents, and solvents such as propylene glycol or glycerin [1]. The e-cigarette was first invented by Herbert A Gilbert in 1963, but the subsequent commercially viable design was patented by Hon Lik of China [2]. E-cigarettes entered the US market in 2007 [3] and have gained tremendous popularity since, especially among youth. E-cigarettes were marketed as a safer alternative to conventional combustible cigarettes and therefore were promoted as harm reduction substitutes for current smokers. However, there are contrasting claims associated with e-cigarettes being considered as a safer alternative to conventional cigarettes. The bottom-line according to the Centers for Disease Control and Prevention remains that e-cigarettes are possibly a less harmful alternative for current smokers addicted to combustible cigarettes [4••]. E-cigarettes are not for people who have never smoked in their life, and even establishing their efficacy as a smoking cessation tool needs thorough and long-term research [5]. The dangers of e-cigarette vaping include damage to the developing brain from nicotine [6–8] and exposure to toxic substances such as heavy metals, volatile organic compounds, and ultrafine particles [9, 10•].

## Public Health Consequences

The adverse health consequences of e-cigarette use for both primary smokers and those exposed to secondhand smoke arises from the inhalation of the e-cigarette aerosol and levels of nicotine delivered into the system [11, 12].

Nicotine is known to be acutely toxic at high doses, and cases of nicotine poisonings due to vape liquids have seen a rise in recent years [13]. Nicotine is also a pharmacologically active biomolecule that sustains addiction, changes the way one’s brain functions [14], and is known to have particularly harmful consequences on the growing fetus if exposed to it during pregnancy [15, 16].

The e-cigarette aerosol contains a vast array of chemicals including any number of approximately 7000 flavorings [17], humectants such as Propylene Glycol and Vegetable Glycerin and contaminants such as metals, formaldehyde, acrolein, and tobacco-specific nitrosamines [1, 18] all with the potential to cause a wide variety of negative health effects. A list of these compounds and their physiologic effects are outlined below (Table 1).

**Table 1:** Constituents of ENDS Aerosol [19]

Serial number	Aerosol component	Health risk	Reference
1.	Ultrafine particles	Asthma, vasoconstriction leading to cardiovascular problems	[20, 21]
2.	Benzene, formaldehyde, acetaldehyde, toluene, cadmium, lead, and nickel	Carcinogen, reproductive toxin	[22, 23]
3.	Propylene glycol	Irritant of the eyes, throat and airways, long-term exposure leads to asthma	[24, 25]
4.	Propylene oxide	Carcinogen	[26]
5.	Diethylene glycol	renal and neurologic toxicity	[27]
6.	Diacetyl and acetyl propionyl (sweet flavorings)	bronchiolitis obliterans	[28, 29]
7.	Carbonyls	Cardiovascular toxicity	[30]
8.	Copper nanoparticles	DNA fragmentation, mitochondrial stress	[31]

## Need for Regulation

In the years before the advent of ENDS technology, various public health measures made significant progress in tobacco control yielding a 6.9% reduction in smoking across the US population from 2005 to 2017 [32–35]. Keeping in mind the economic and social burden exacted by smoking-related diseases, the Federal Government had enacted various laws to make the sale of conventional tobacco products more difficult especially for the younger generation. However, rapid and unchecked increase in e-cigarette use [10•, 36] has once again threatened to endanger the health of our youth through nicotine addiction and vaping related disease [6, 37]. The National Youth Tobacco Survey held jointly by the FDA and the Centers for Disease Control and Prevention shows that around 3.6 million students (both middle and high school) were using e-cigarettes in 2018, up from 2.1 million in 2017 [38, 39]. There is emerging evidence that e-cigarettes can spur future tobacco product use in teens, whereas, on the other hand, banning tobacco products diminishes the smoking risk. Socio-economic background is another factor that also plays a major role in smoking initiation [40–42].

Another area of concern is the public health consequences of secondhand e-cigarette smoke on bystanders. Though the country has made significant progress in enacting clean air laws in public places including workplaces and indoors, a lot still remains to be done. The use of e-cigarettes in public areas poses a serious health risk considering the various toxic constituents that have been shown to affect both the primary smoker and victims of passive smoking. It is pertinent to note that smoke-free laws in the USA were passed before ENDS entered the market and do not specifically mention the prohibition of e-cigarette smoking in many places. As such, this non-clarity may lead to non-compliance or exploitation of smoke-free rules [43, 44].

## Existing Regulation

### Federal Regulations

The FDA has been regulating tobacco products since June 2009; a timeline of the most important regulations is furnished in Table 2.

**Table 2:** Timeline of policies/rules/regulations enforced at the federal level

No.	Date	Name of agency	Regulation particulars	Implication
1.	June, 2009	Food and Drug Administration (FDA) of the Department of Health and Human Services.	Family Smoking Prevention and Tobacco Control Act. [45]	Authorizing FDA to regulate tobacco products including e-cigarettes. It led to the creation of center for tobacco products.
2.	April, 2014	Food and Drug Administration (FDA) of the Department of Health and Human Services.	Proposed Deeming Regulations [46]	Authorized the FDA to put heavy restrictions on most of the existing unregulated e-cigarette manufacturing industry and required premarket tobacco applications (PMTA’s) for new manufacturers

On May 10, 2016, the U.S. Food and Drug Administration (FDA) passed a new rule effective August 08, 2016, deeming that all tobacco products be brought under the purview of Federal Food, Drug, and Cosmetic Act thus authorizing the FDA to regulate all tobacco products including ENDS [46]. Apart from banning the sale of e-cigarettes to those below 18 years of age, the rule also stipulates several manufacturing standards and marketing limitations. The rule focuses on preventing a younger generation from becoming addicted to nicotine through e-cigarettes while taking into account the harm reduction potential of e-cigarettes for existing smokers addicted to nicotine.

More recently, in light of the 2018 National Youth Tobacco Survey, the FDA and Federal Trade Commission (FTC) issued warnings to four e-cigarette manufacturing companies around youth-focused advertisement, sale and distribution of ENDS products, especially on social media platforms [47].

### State Regulations of e-Cigarettes

The US state and local governments have played a proactive role in enacting several laws at their level to protect against the misuse of e-cigarettes. In June 2019, San Francisco, California, became the first city in the USA to ban the retail and online sale of e-cigarettes. This move is especially significant as Juul Labs, Inc., the makers of the Juul e-cigarettes variety, which has captured 70% of ENDS market share in recent years, is based out of San Francisco. Another trend is the implementation of Tobacco 21 laws in several states, increasing the minimum age of sale of tobacco products from 18 to 21. As of June 2019, 16 states, Arkansas, California, Connecticut, Delaware, Hawaii, Illinois, Maine, Maryland, Massachusetts, New Jersey, Oregon, Texas, Utah, Vermont, Virginia, and Washington, the District of Columbia and 470 localities had implemented tobacco 21 laws [48].

As of April 2019, 13 states, 2 territories, and 841 municipalities have banned the use of e-cigarettes in 100% smoke-free public places [43]. In addition, regulations defining e-cigarettes, taxation, packaging, access to youth, and licensure of e-cigarette sales have been put into place across several states (Table 3).

**Table 3:** Law(s) in effect across all States & the District of Columbia in the USA (March 15, 2019) [49]

	State	Law(s) that define e-cigarettes	Law(s) taxing e-cigarettes	Law(s) on product packaging of e-cigarettes	Law(s) restricting youth access to e-cigarettes	Law(s) requiring licenses for retail sales of e-cigarettes
1	Alabama				Yes	
2	Alaska				Yes	Yes
3	Arizona				Yes	
4	Arkansas			Yes	Yes	Yes
5	California	Yes	Yes	Yes	Yes	Yes
6	Colorado	Yes			Yes	
7	Connecticut				Yes	Yes
8	Delaware	Yes	Yes		Yes	Yes (vape liquid)
9	District of Columbia	Yes	Yes		Yes	Yes
10	Florida				Yes	
11	Georgia				Yes	
12	Hawaii	Yes			Yes	Yes
13	Idaho				Yes	
14	Illinois			Yes	Yes	
15	Indiana	Yes		Yes	Yes	Yes
16	Iowa				Yes	Yes
17	Kansas		Yes		Yes	Yes
18	Kentucky				Yes	
19	Louisiana		Yes		Yes	Yes
20	Maine	Yes		Yes	Yes	Yes
21	Maryland				Yes	Yes
22	Massachusetts	Yes		Yes	Yes	
23	Michigan					
24	Minnesota	Yes	Yes	Yes	Yes	Yes
25	Mississippi				Yes	
26	Missouri			Yes	Yes	Yes
27	Montana				Yes	Yes
28	Nebraska				Yes	
29	Nevada				Yes	
30	New Hampshire			Yes	Yes	
31	New Jersey	Yes	Yes	Yes	Yes	
32	New Mexico			Yes	Yes	
33	New York			Yes	Yes	
34	North Carolina	Yes	Yes	Yes	Yes	Yes (non-local manufacturers)
35	North Dakota			Yes	Yes	
36	Ohio			Yes	Yes	
37	Oklahoma				Yes	
38	Oregon			Yes	Yes	
39	Pennsylvania	Yes	Yes	Yes		Yes
40	Rhode Island			Yes	Yes	Yes
41	South Carolina				Yes	
42	South Dakota	Yes		Yes	Yes	
43	Tennessee			Yes	Yes	
44	Texas			Yes	Yes	Yes
45	Utah	Yes		Yes	Yes	Yes
46	Vermont			Yes	Yes	Yes
47	Virginia			Yes	Yes	
48	Washington			Yes	Yes	Yes
49	West Virginia	Yes	Yes		Yes	
50	Wisconsin				Yes	
51	Wyoming	Yes		Yes	Yes	

## Challenges and Recommendations

A review of the scientific literature shows largely incomplete data around the health effects of e-cigarettes. This translates to policy indecision among the regulatory authorities leading to confusion among the general public. This also causes problems for health care professionals in counseling current smokers looking to switch to e-cigarettes [50–52]. A long-term comprehensive study involving all major stakeholders is required to address this problem.

A bi-pronged approach could be key in balancing the regulatory aspects of e-cigarettes. It should consist of a prevention strategy in case of youth and a control strategy for current smokers who are looking at reduced harm alternatives for their nicotine fixation [53]. The prevention aspect can include laws that prohibit sale to minors, prevent youth-targeted advertisement campaigns [54, 55] and flavors, child-safe packaging, and campaigns addressing awareness and education. On the other hand, the control aspect can include better manufacturing measures, licensing laws for retail and online sale, selective taxation, and supervised subsidy for verified current smokers.

## Conclusions

Every once in a while, a newer technology emerges onto the market and causes a massive shift in the prevailing status quo. E-cigarettes are one such technology that emerged a decade ago and has changed the way tobacco is consumed by the current population. It has brought along with it many dangers but also some promises. The regulatory framework has to tread a narrow path of prevention and control to safeguard future generation against the evils of tobacco as well as other unintended health consequences of using ENDS, but at the same time ensure that the path from combustible cigarettes to e-cigarettes ends with complete smoking cessation.
